# Anatomic assessment of pulmonary veins and left atrium - comparison of magnetic resonance angiography with gadoteric acid, the blood pool contrast agent B22956/1 and a non-contrast enhanced imaging approach

**DOI:** 10.1186/1532-429X-13-S1-P260

**Published:** 2011-02-02

**Authors:** Szilard Rona, Volker Rasche, Georg Grossmann, Sascha Stiller, Nico Merkle

**Affiliations:** 1 University of Ulm, Ulm, Germany

## Introduction

Pulmonary vein isolation (PVI) is the utmost method to treat patients suffering from symptomatic drug-refractory atrial fibrillation (AF). Non-invasive imaging of the left atrium (LA) and pulmonary veins prior to PVI plays an important role in procedural planning and guiding.

## Purpose

To compare the feasibility and image quality of three magnetic resonance angiographic methods using the blood pool contrast agent B22956/1 (Bracco), the extravascular contrast agent gadoteric acid (Dotarem®, Guerbet) and a non-contrast protocol to evaluate the left atrium and pulmonary veins.

## Methods

We compared 3 different MRI methods. First a non-contrast enhanced T2-prep steady state free precession (SSFP)-sequence, second, a high resolution IR-SSFP sequence with the blood pool contrast agent B22956/1 and third, a breath hold IR-SSFP atriography using the extravascular contrast agent gadoteric acid. Fourteen healthy volunteers (B22956/1-IR-SSFP) and 20 consecutive patients (T2-prep SSFP-sequence and IR-SSFP sequence with gadoteric acid) with history of paroxysmal AF scheduled for PVI were involved in the study. Signal-to-noise ratio (SNR) of blood in the left atrium and contrast-to-noise ratio (CNR) between left atrial blood and myocardium was assessed in all of the three groups. Furthermore, image quality was assessed by two observers.

## Results

Regarding SNR there was no significant difference among the three sequences (13,7±3,1 for B22956/1 vs. 11,2±3,2 for gadoteric acid vs. 11,4±4,3 for T2-prep-SSFP group, respectively). CNR significantly improved for B22956/1 compared with T2-prep-SSFP (14,8±3,6 vs. 7,1±3,6; p<0,0001) and for gadoteric acid compared with T2-prep-SSFP (14,4±4,1 vs. 7,1±3,6; p<0,0001). There was no significant difference regarding CNR between the two contrast-enhanced methods (14,8±3,6 for B22956/1 vs. 14,4±4,1 for gadoteric acid). Regarding acquisition times the gadoteric acid method was significantly less time consuming compared with the B22956/1 (15,4±1,2 sec. vs. 508,1±113,8 sec. p<0,0001) and the non-contrast method (15,4±1,2s vs. 194,6±44,7s, p<0,0001). Image quality for assessment and reconstruction of atrium and PV was best for the blood pool agent, followed by gadoteric acid. In contrast, sufficient imaging of PVs was not adequate in several patients with the non-contrast sequence.

## Conclusion

Gadoteric acid-enhanced and B22956/1-enhanced MRI of the left atrium significantly improved signal and image quality compared with non-contrast MRI. Regarding availability, costs as well as acquisition times the breath hold IR-SSFP with gadoteric acid seems to be the most appropriate.

